# Supplementary material for: Genotype by Environment Interaction and Selection Response for Milk Yield Traits and Conformation in a Local Cattle Breed Using a Reaction Norm Approach
Source: Animals (Basel). 2022 Mar 26;12(7):839. doi: 10.3390/ani12070839 (PMC8996846; doi:10.3390/ani12070839)
Supplement: Supplementary file 1 [file animals-12-00839-s001.zip › animals-1621646-supplementary.pdf]

Supplementary Table S1. Number of levels for the HEG effect in the two datasets under study (milk yield traits, MT and linear type traits and factor scores, LTT/FS), and number of the records within each level of the environmental categories considered in the study. Environmental groups considered for statistics are bolded.

	MT		LTT/FS	
	No. levels HEG	No. records	No. levels HEG	No. records
Environmental categories, or EC				
Geographical area				
Plain	10	108,645	52	5,783
Hill	57	5,339	9	247
Mountain	55	49,875	48	2,508
Housing				
Tie-Stall	99	110,713	86	5,963
Loose Housing	23	53,146	23	2,575
Feeding				
Traditional	96	108,566	88	5,806
TMR	26	55,293	21	2,732
Pasture				
No	62	97,052	57	5,091
Yes	60	66,807	52	3,447
Environmental groups, or EG[1]				
**Plain_TS_Trad_No**	**25**	**50,969**	**25**	**2,769**
**Plain_TS_Trad_Yes**	**10**	**13,936**	**8**	**786**
Plain_LH_Trad_No	3	3,013	3	179
**Plain_TS_TMR_No**	**9**	**11,490**	**7**	**629**
Plain_TS_TMR_Yes	1	551	1	30
**Plain_LH_TMR_No**	**7**	**22,418**	**6**	**1,008**
Plain_LH_TMR_Yes	2	6,268	2	382
Hill_TS_Trad_No	2	828	2	39
Hill_TS_Trad_Yes	6	3,132	5	141
Hill_TS_TMR_Yes	1	1,323	1	65
Hill_LH_TMR_No	1	56	1	2
Mountain_TS_Trad_No	8	1,798	7	112
**Mountain_TS_Trad_Yes**	**34**	**23,934**	**29**	**1,234**
**Mountain_LH_Trad_No**	**6**	**6,304**	**5**	**339**
Mountain_LH_Trad_Yes	2	4,652	2	207
Mountain_TS_TMR_No	1	176	1	14
Mountain_TS_TMR_Yes	2	2,576	2	144
Mountain_LH_TMR_Yes	2	10,435	2	458

[1]TS=Tie Stall; LH=Loose Housing; Trad=Traditional; TMR=Total Mixed Ration; Yes=Occurrence of pasture: yes; No=Occurrence of pasture: no. 2

Supplementary Table S2. Phenotypic means (SD in brackets) within levels of environment categories for traits considered in the study.

	Geographical area			Type of housing		Feeding system		Summer pasture	
Environment	Plain	Hill	Mountain	Tie-stall	Loose housing	Traditional	TMR[1]	No	Yes
Milk productive traits									
No.	108645	5339	49875	110713	53146	108566	55293	97052	66807
Milk yield (kg)	17.76 (5.931)	15.14 (4.769)	16.43 (5.261)	16.48 (5.381)	18.91 (6.121)	16.73 (5.49)	18.34 (6.075)	17.98 (5.861)	16.25 (5.411)
Fat yield (kg)	0.621 (0.217)	0.529 (0.178)	0.575 (0.205)	0.575 (0.198)	0.665 (0.232)	0.581 (0.202)	0.649 (0.229)	0.63 (0.215)	0.566 (0.206)
Fat %	3.525 (0.556)	3.535 (0.601)	3.521 (0.622)	3.516 (0.574)	3.541 (0.587)	3.504 (0.572)	3.564 (0.588)	3.536 (0.554)	3.507 (0.611)
Protein yield (kg)	0.591 (0.191)	0.482 (0.145)	0.541 (0.168)	0.541 (0.17)	0.638 (0.198)	0.549 (0.175)	0.619 (0.195)	0.6 (0.188)	0.533 (0.173)
Protein %	3.364 (0.353)	3.221 (0.343)	3.32 (0.342)	3.319 (0.356)	3.402 (0.332)	3.312 (0.352)	3.412 (0.338)	3.371 (0.352)	3.31 (0.345)
SCS	2.879 (1.821)	2.787 (1.771)	2.649 (2.031)	2.869 (1.912)	2.676 (1.833)	2.779 (1.922)	2.859 (1.821)	2.776 (1.844)	2.849 (1.951)
Linear type traits									
No.	5783	247	2508	5963	2575	5806	2732	5091	3447
Body size	3.151 (0.864)	2.745 (0.762)	3.132 (0.857)	3.129 (0.868)	3.143 (0.849)	3.16 (0.873)	3.077 (0.836)	3.15 (0.852)	3.109 (0.876)
Muscularity	2.958 (0.712)	2.757 (0.758)	2.873 (0.661)	2.9 (0.722)	2.988 (0.644)	2.882 (0.7)	3.022 (0.693)	2.978 (0.706)	2.852 (0.687)
Body shape	2.885 (0.822)	2.899 (0.823)	2.966 (0.833)	2.848 (0.827)	3.052 (0.806)	2.89 (0.829)	2.951 (0.819)	2.876 (0.826)	2.958 (0.824)
Udder	3.143 (0.934)	3.142 (0.749)	3.087 (0.935)	3.074 (0.913)	3.248 (0.955)	3.111 (0.927)	3.159 (0.935)	3.161 (0.939)	3.075 (0.914)
Stature	3.157 (0.939)	2.652 (0.86)	3.13 (0.927)	3.134 (0.946)	3.134 (0.917)	3.175 (0.947)	3.048 (0.911)	3.15 (0.931)	3.111 (0.946)
Body length	3.196 (0.904)	2.777 (0.818)	3.217 (0.899)	3.187 (0.908)	3.198 (0.892)	3.222 (0.911)	3.122 (0.883)	3.199 (0.895)	3.178 (0.915)
Thorax depth	3.206 (0.869)	2.98 (0.788)	3.174 (0.882)	3.19 (0.88)	3.191 (0.852)	3.209 (0.884)	3.149 (0.844)	3.211 (0.861)	3.159 (0.886)
Thorax length	3.08 (0.787)	2.777 (0.689)	2.985 (0.767)	3.022 (0.786)	3.092 (0.767)	3.035 (0.786)	3.062 (0.768)	3.101 (0.776)	2.959 (0.78)
Shoulder, Fore view	2.801 (0.794)	2.603 (0.814)	2.594 (0.755)	2.702 (0.795)	2.81 (0.77)	2.668 (0.78)	2.874 (0.791)	2.825 (0.784)	2.6 (0.779)
Back, Loins and Rump	2.936 (0.741)	2.652 (0.791)	2.833 (0.678)	2.855 (0.743)	2.996 (0.681)	2.833 (0.723)	3.035 (0.718)	2.958 (0.726)	2.808 (0.72)
Thigh, Buttocks side view	3.022 (0.738)	2.773 (0.805)	2.942 (0.682)	2.967 (0.748)	3.048 (0.666)	2.951 (0.729)	3.077 (0.711)	3.047 (0.729)	2.909 (0.713)
Thigh, Buttocks rear view	2.82 (0.774)	2.684 (0.774)	2.756 (0.72)	2.77 (0.778)	2.862 (0.708)	2.755 (0.758)	2.888 (0.754)	2.84 (0.769)	2.735 (0.739)
Thinness	3.22 (0.777)	3.417 (0.744)	3.276 (0.764)	3.221 (0.787)	3.291 (0.74)	3.238 (0.771)	3.251 (0.779)	3.223 (0.778)	3.271 (0.766)
Rump angle	2.741 (0.588)	2.789 (0.574)	2.747 (0.611)	2.715 (0.606)	2.812 (0.559)	2.74 (0.592)	2.754 (0.598)	2.741 (0.594)	2.749 (0.594)
Rump width	3.155 (0.807)	2.939 (0.687)	3.134 (0.817)	3.14 (0.81)	3.148 (0.802)	3.166 (0.817)	3.093 (0.785)	3.153 (0.799)	3.126 (0.82)
Rear legs side view	3.141 (0.786)	3.004 (0.713)	3.187 (0.749)	3.106 (0.795)	3.254 (0.71)	3.135 (0.777)	3.184 (0.766)	3.175 (0.785)	3.115 (0.755)
Feet	2.879 (0.672)	2.826 (0.667)	2.918 (0.544)	2.826 (0.63)	3.034 (0.629)	2.854 (0.619)	2.963 (0.666)	2.87 (0.667)	2.916 (0.589)
Fore udder attach	3.331 (0.961)	3.198 (0.918)	3.234 (0.954)	3.262 (0.954)	3.384 (0.966)	3.275 (0.965)	3.347 (0.944)	3.338 (0.966)	3.24 (0.946)
Rear udder attach	3.024 (0.903)	3.113 (0.788)	3.057 (0.881)	3.007 (0.891)	3.102 (0.897)	3.042 (0.889)	3.023 (0.903)	3.032 (0.906)	3.041 (0.875)
Udder width	3.095 (0.957)	3.113 (0.853)	2.995 (0.958)	3.031 (0.946)	3.149 (0.971)	3.065 (0.954)	3.068 (0.958)	3.123 (0.948)	2.982 (0.96)
Udder depth	3.299 (0.657)	3.32 (0.617)	3.39 (0.66)	3.34 (0.667)	3.294 (0.637)	3.34 (0.662)	3.296 (0.648)	3.264 (0.652)	3.418 (0.657)
Suspensory ligament	3.184 (0.801)	3.316 (0.914)	3.246 (0.768)	3.147 (0.784)	3.343 (0.804)	3.175 (0.792)	3.271 (0.798)	3.175 (0.802)	3.251 (0.783)
Teat placement side	3.061 (0.734)	2.891 (0.716)	2.936 (0.75)	2.977 (0.745)	3.117 (0.72)	2.987 (0.747)	3.087 (0.723)	3.054 (0.736)	2.968 (0.745)
Teat length	3.055 (0.805)	2.984 (0.791)	3.143 (0.823)	3.09 (0.827)	3.052 (0.773)	3.114 (0.815)	3.004 (0.798)	3.041 (0.813)	3.134 (0.806)
Factor scores[2]									
Muscularity factor	0.018 (0.964)	-0.292 (1.021)	-0.145 (0.879)	-0.086 (0.967)	0.068 (0.884)	-0.116 (0.94)	0.124 (0.936)	0.049 (0.948)	-0.17 (0.926)
Udder conformation factor	-0.047 (0.944)	0.068 (0.987)	0.008 (0.973)	-0.059 (0.966)	0.046 (0.922)	-0.059 (0.961)	0.039 (0.936)	-0.073 (0.952)	0.04 (0.953)
Udder volume factor	0.073 (1)	0.064 (0.881)	-0.018 (0.992)	-0.006 (0.982)	0.166 (1.015)	0.03 (0.997)	0.081 (0.992)	0.1 (0.998)	-0.034 (0.986)

[1]Total Mixed Ration; [2]No. is the same as for linear type traits

Supplementary Table S3. Variance components (posterior means and SE in brackets) estimated from sire model not including GxE.

Trait	Variances[1]			
	H	Pe	G	R
Milk	1.862 (0.037)	5.75 (0.094)	0.814 (0.092)	6.655 (0.026)
Fat yield	2.941 (0.062)	7.369 (0.126)	0.94 (0.115)	13.844 (0.053)
Fat %	0.045 (0.001)	0.054 (0.001)	0.011 (0.001)	0.195 (0.001)
Protein yield	2.668 (0.049)	5.385 (0.09)	0.704 (0.084)	7.279 (0.029)
Protein %	0.012 (0.0002)	0.024 (0.0004)	0.005 (0.001)	0.044 (0.0002)
SCS	0.198 (0.005)	0.854 (0.015)	0.066 (0.009)	1.968 (0.007)
Muscularity (factor)	0.051 (0.007)		0.049 (0.007)	0.636 (0.011)
Udder conform.	0.031 (0.006)		0.047 (0.008)	0.772 (0.014)
Udder volume	0.014 (0.005)		0.073 (0.01)	0.798 (0.013)
Body size	0.006 (0.003)		0.058 (0.008)	0.586 (0.01)
Muscularity (linear)	0.021 (0.003)		0.023 (0.004)	0.379 (0.007)
Body shape	0.012 (0.004)		0.023 (0.005)	0.594 (0.01)
Udder	0.003 (0.002)[3]		0.061 (0.009)	0.734 (0.012)
Stature	0.007 (0.004)		0.081 (0.01)	0.692 (0.012)
Body length	0.006 (0.003)		0.05 (0.007)	0.654 (0.011)
Thorax depth	0.012 (0.004)		0.042 (0.006)	0.615 (0.01)
Thorax length	0.016 (0.003)		0.022 (0.004)	0.483 (0.008)
Shoulder, Fore view	0.035 (0.004)		0.031 (0.005)	0.449 (0.008)
Back, Loins and Rump	0.028 (0.004)		0.023 (0.004)	0.404 (0.007)
Thigh, Buttocks side	0.024 (0.004)		0.026 (0.004)	0.41 (0.007)
Thigh, Buttocks rear	0.027 (0.004)		0.031 (0.005)	0.438 (0.008)
Thinness	0.01 (0.003)		0.042 (0.007)	0.529 (0.009)
Rump angle	0.004 (0.002)		0.03 (0.004)	0.301 (0.005)
Rump width	0.024 (0.004)		0.029 (0.005)	0.485 (0.008)
Rear legs side view	0.013 (0.004)		0.043 (0.006)	0.503 (0.009)
Feet	0.013 (0.003)		0.009 (0.002)	0.352 (0.006)
Fore udder attach	0.021 (0.005)		0.059 (0.009)	0.735 (0.012)
Rear udder attach	0.014 (0.004)		0.037 (0.006)	0.687 (0.012)
Udder width	0.006 (0.004)[3]		0.073 (0.01)	0.774 (0.013)
Udder depth	0.021 (0.003)		0.021 (0.003)	0.366 (0.006)
Suspensory ligament	0.023 (0.004)		0.013 (0.003)	0.536 (0.009)
Teat placement side	0.009 (0.003)		0.04 (0.005)	0.465 (0.008)
Teat length	0.006 (0.003)		0.044 (0.006)	0.576 (0.01)

[1]H = Variance of herd-test-day (*htd*) or herd-year-classifier (*hyc*) effect, depending on the dataset; Pe = Permanent environmental variance; G = genetic variance; GxE = genetic by environmental variance; covG,GxE = covariance between genetic and GxE components; R = residual variance, calculated as average of the 5 residual variance estimates. [2]Variances have been multiplied by 10^3. [3]Variances not significantly different from zero (z-scores test). The other variances in table are significantly different from zero ($P \le 0.05$)

Supplementary Table S4. Average accuracies with standard deviations in brackets of bulls' EBVs obtained from the sire reaction norm model including GxE (*acc*_GxE) and from the reaction norm model without GxE (*acc*_noGxE). Milk yield traits: n = 807; Linear type traits/Factor scores: n = 863.

Trait	*acc*_GxE	*acc*_noGxE
Milk	0.548 (0.281)	0.583 (0.296)
Fat yield	0.543 (0.287)	0.567 (0.292)
Fat %	0.591 (0.296)	0.594 (0.3)
Protein yield	0.536 (0.281)	0.573 (0.293)
Protein %	0.6 (0.298)	0.606 (0.301)
SCS	0.489 (0.263)	0.517 (0.278)
Muscularity (factor)	0.472 (0.274)	0.504 (0.276)
Udder conform.	0.455 (0.264)	0.471 (0.263)
Udder volume	0.497 (0.283)	0.508 (0.277)
Body size	0.498 (0.285)	0.522 (0.285)
Muscularity (linear)	0.453 (0.267)	0.485 (0.268)
Body shape	0.419 (0.247)	0.432 (0.242)
Udder	0.497 (0.284)	0.507 (0.276)
Stature	0.52 (0.295)	0.535 (0.289)
Body length	0.484 (0.279)	0.499 (0.275)
Thorax depth	0.416 (0.244)	0.484 (0.269)
Thorax length	0.42 (0.249)	0.445 (0.251)
Shoulder, Fore view	0.47 (0.274)	0.482 (0.267)
Back, Loins and Rump	0.451 (0.265)	0.479 (0.267)
Thigh, Buttocks side	0.451 (0.265)	0.495 (0.271)
Thigh, Buttocks rear	0.472 (0.274)	0.491 (0.269)
Thinness	0.491 (0.284)	0.504 (0.278)
Rump angle	0.466 (0.269)	0.499 (0.274)
Rump width	0.45 (0.263)	0.476 (0.263)
Rear legs side view	0.479 (0.276)	0.493 (0.273)
Feet	0.265 (0.181)	0.349 (0.207)
Fore udder attach	0.471 (0.271)	0.49 (0.27)
Rear udder attach	0.44 (0.258)	0.456 (0.255)
Udder width	0.506 (0.289)	0.517 (0.28)
Udder depth	0.427 (0.254)	0.451 (0.252)
Suspensory ligament	0.38 (0.23)	0.392 (0.225)
Teat placement side	0.458 (0.263)	0.497 (0.273)
Teat length	0.455 (0.269)	0.482 (0.266)

Supplementary Table S5. Least square means with standard error (SE) of sire model heritability in different levels of the target environmental categories (EC: Geographical area; Housing; Feeding; Occurrence of pasture). The significance of difference among the levels of the same EC has been tested via GLM analysis.

Trait	Geographical area					Housing				Feeding				Pasture			
	Plain	Hill	Mountain	SE	*P*	Tie-Stall	Loose H.[1]	SE	*P*	Traditional	TMR[2]	SE	*P*	No	Yes	SE	*P*
Milk yield	0.271	0.269	0.263	0.011		0.266	0.272	0.009		0.267	0.269	0.008		0.272	0.263	0.007	
Fat yield	0.181	0.149	0.155	0.008	**	0.162	0.186	0.007	*	0.164	0.175	0.007		0.179	0.154	0.005	**
Fat %	0.149	0.154	0.147	0.002		0.148	0.149	0.002		0.148	0.149	0.002		0.150	0.147	0.001	
Protein yield	0.229	0.209	0.208	0.012		0.214	0.240	0.010		0.217	0.221	0.009		0.232	0.204	0.007	**
Protein %	0.240	0.227	0.234	0.006		0.233	0.248	0.005	*	0.232	0.251	0.004	**	0.241	0.231	0.004	
SCS	0.100	0.089	0.102	0.004		0.099	0.103	0.003		0.100	0.099	0.003		0.103	0.097	0.003	
Muscularity (factor)	0.288	0.240	0.248	0.018		0.259	0.295	0.014		0.252	0.318	0.014	**	0.289	0.242	0.012	**
Udder conformation	0.246	0.259	0.238	0.007		0.246	0.234	0.005		0.245	0.239	0.005		0.250	0.237	0.004	*
Udder volume	0.346	0.385	0.322	0.014	*	0.334	0.359	0.011		0.339	0.334	0.010		0.340	0.337	0.009	
Body size	0.380	0.369	0.383	0.008		0.382	0.378	0.006		0.385	0.366	0.006	*	0.379	0.383	0.005	
Muscularity (linear)	0.242	0.192	0.212	0.015		0.220	0.241	0.012		0.213	0.267	0.011	**	0.239	0.209	0.010	*
Body shape	0.160	0.162	0.169	0.009		0.158	0.188	0.007	**	0.163	0.168	0.007		0.161	0.168	0.006	
Udder	0.313	0.323	0.289	0.018		0.295	0.342	0.014	*	0.302	0.308	0.013		0.312	0.294	0.011	
Stature	0.441	0.446	0.443	0.008		0.445	0.434	0.006		0.446	0.430	0.006		0.441	0.445	0.005	
Body length	0.298	0.300	0.306	0.004		0.304	0.289	0.003	**	0.303	0.295	0.003		0.298	0.305	0.003	
Thorax depth	0.268	0.257	0.262	0.005		0.265	0.264	0.004		0.266	0.260	0.004		0.266	0.263	0.003	
Thorax length	0.177	0.188	0.187	0.004		0.184	0.176	0.004		0.185	0.174	0.003	*	0.179	0.186	0.003	
Shoulder, Fore view	0.272	0.224	0.215	0.017	**	0.236	0.270	0.014		0.228	0.295	0.013	**	0.269	0.213	0.011	***
Back, Loins and Rump	0.224	0.172	0.188	0.018		0.197	0.235	0.014		0.188	0.263	0.013	***	0.227	0.180	0.012	**
Thigh, Buttocks side view	0.255	0.230	0.239	0.008		0.243	0.255	0.007		0.241	0.264	0.006	*	0.255	0.235	0.005	*
Thigh, Buttocks rear view	0.271	0.255	0.247	0.016		0.253	0.284	0.013		0.249	0.296	0.012	*	0.270	0.247	0.011	
Thinness	0.291	0.330	0.301	0.008	*	0.298	0.299	0.006		0.301	0.290	0.006		0.294	0.303	0.005	
Rump angle	0.380	0.354	0.386	0.024		0.371	0.421	0.018		0.388	0.356	0.018		0.376	0.386	0.015	
Rump width	0.239	0.239	0.234	0.008		0.236	0.240	0.006		0.240	0.227	0.006		0.240	0.234	0.005	
Rear legs side view	0.359	0.365	0.349	0.019		0.361	0.331	0.015		0.350	0.372	0.014		0.350	0.361	0.012	
Feet	0.140	0.116	0.131	0.012		0.137	0.123	0.009		0.137	0.124	0.009		0.141	0.127	0.008	
Fore udder attach	0.298	0.309	0.262	0.015	*	0.277	0.307	0.012		0.280	0.295	0.011		0.293	0.272	0.010	
Rear udder attach	0.229	0.270	0.228	0.009	*	0.232	0.226	0.007		0.230	0.237	0.007		0.226	0.237	0.006	
Udder width	0.359	0.399	0.321	0.016	**	0.341	0.360	0.013		0.347	0.337	0.013		0.355	0.334	0.011	
Udder depth	0.253	0.283	0.269	0.013		0.265	0.251	0.010		0.268	0.244	0.010		0.261	0.265	0.008	
Suspensory ligament	0.103	0.111	0.106	0.004		0.105	0.105	0.003		0.105	0.105	0.003		0.108	0.102	0.002	
Teat placement side	0.332	0.322	0.305	0.010	*	0.315	0.336	0.008		0.317	0.327	0.008		0.329	0.308	0.006	*
Teat length	0.287	0.295	0.297	0.005		0.291	0.293	0.004		0.293	0.286	0.004		0.289	0.294	0.003	

[1]Loose Housing; [2]Total Mixed Ration; *= $P \leq 0.05$; **= $P \leq 0.01$: ***= $P \leq 0.001$

Supplementary Table S6. Least square means with standard error (SE) of sire model heritability in different environmental groups[1] considered in the study. The significance of difference among the levels of the same EC has been tested via GLM analysis.

Trait	Environmental groups[1]						SE	*P*
	Mountain TS Trad Yes	Mountain LH Trad No	Plain TS Trad No	Plain TS TMR No	Plain TS Trad Yes	Plain LH TMR No		
Milk yield	0.260	0.279	0.258	0.305	0.281	0.267	0.023	
Fat yield	0.152	0.189	0.179	0.189	0.167	0.189	0.018	
Fat %	0.147	0.155	0.151	0.148	0.147	0.151	0.004	
Protein yield	0.203	0.246	0.222	0.250	0.218	0.242	0.026	
Protein %	0.233	0.234	0.230	0.249	0.224	0.283	0.011	***
SCS	0.102	0.119	0.099	0.110	0.091	0.091	0.009	
Muscularity (factor)	0.235	0.282	0.272	0.353	0.264	0.338	0.037	
Udder conformation	0.236	0.261	0.255	0.250	0.232	0.233	0.012	
Udder volume	0.317	0.380	0.350	0.315	0.355	0.363	0.026	
Body size	0.383	0.399	0.379	0.372	0.402	0.365	0.016	
Muscularity (linear)	0.208	0.218	0.228	0.292	0.223	0.293	0.031	
Body shape	0.167	0.216	0.150	0.148	0.171	0.187	0.018	*
Udder	0.280	0.356	0.320	0.283	0.291	0.347	0.035	
Stature	0.443	0.442	0.440	0.439	0.451	0.424	0.015	
Body length	0.304	0.302	0.295	0.311	0.314	0.278	0.008	*
Thorax depth	0.259	0.270	0.267	0.268	0.282	0.260	0.009	
Thorax length	0.184	0.173	0.177	0.170	0.186	0.172	0.007	
Shoulder, Fore view	0.207	0.249	0.258	0.330	0.242	0.330	0.035	**
Back, Loins and Rump	0.175	0.211	0.208	0.303	0.182	0.279	0.036	*
Thigh, Buttocks side view	0.236	0.248	0.252	0.275	0.235	0.285	0.018	
Thigh, Buttocks rear view	0.245	0.279	0.253	0.316	0.266	0.322	0.033	
Thinness	0.296	0.310	0.288	0.279	0.307	0.302	0.013	
Rump angle	0.379	0.400	0.390	0.291	0.369	0.402	0.046	
Rump width	0.235	0.252	0.243	0.227	0.241	0.241	0.018	
Rear legs side view	0.361	0.291	0.329	0.437	0.391	0.337	0.039	
Feet	0.127	0.135	0.155	0.123	0.133	0.112	0.025	
Fore udder attach	0.250	0.310	0.294	0.297	0.302	0.312	0.029	
Rear udder attach	0.226	0.223	0.229	0.231	0.215	0.210	0.014	
Udder width	0.320	0.411	0.377	0.319	0.351	0.380	0.032	
Udder depth	0.263	0.282	0.259	0.249	0.258	0.246	0.026	
Suspensory ligament	0.103	0.109	0.104	0.109	0.100	0.102	0.007	
Teat placement side	0.300	0.314	0.326	0.329	0.327	0.350	0.019	
Teat length	0.299	0.312	0.286	0.286	0.287	0.285	0.010	

[1]TS=Tie-Stall; LH=Loose Housing; Trad=Traditional; TMR=Total Mixed Ration; Yes=Occurrence of pasture: yes; No=Occurrence of pasture: no; *= $P \leq 0.005$; **= $P \leq 0.01$: ***= $P \leq 0.001$

Supplementary Table S7. Multivariate response to selection for traits included in Rendena selection index by levels of environmental categories (A) and by environmental groups (B). Values of response are shown by generation and expressed in the unit of measurement of the respective trait.

A)

Trait	Geographical area			Housing		Feeding		Pasture	
	Plain	Hill	Mountain	Tie-Stall	Loose H.[1]	Traditional	TMR[2]	No	Yes
Milk (kg)	1.574	1.662	1.348	1.373	1.912	1.432	1.653	1.610	1.341
Fat (kg)	0.052	0.047	0.040	0.043	0.062	0.044	0.057	0.053	0.040
Protein (kg)	0.060	0.055	0.047	0.049	0.074	0.051	0.063	0.061	0.046
Muscularity (factor, pts)	0.005	-0.010	0.021	0.019	-0.017	0.015	-0.004	0.002	0.021
Udder Conformation (pts)	-0.033	-0.040	-0.029	-0.029	-0.042	-0.031	-0.034	-0.035	-0.029
Udder Volume (pts)[3]	0.205	0.344	0.177	0.187	0.270	0.192	0.244	0.219	0.186
ADG (kg/d)	0.040	0.039	0.042	0.042	0.039	0.041	0.040	0.040	0.042
SEUROP (pts)	1.312	1.270	1.401	1.390	1.208	1.365	1.284	1.297	1.408
Dressing % (pts)	0.239	0.245	0.262	0.258	0.219	0.253	0.233	0.236	0.264
SCS (points)	0.241	0.227	0.262	0.252	0.241	0.252	0.237	0.241	0.258

B)

Trait	Environmental groups[4]					
	Mountain TS Trad Yes	Mountain LH Trad No	Plain TS Trad No	Plain TS TMR No	Plain TS Trad Yes	Plain LH TMR No
Milk (kg)	1.344	1.542	1.392	1.593	1.444	1.975
Fat (kg)	0.039	0.051	0.047	0.056	0.044	0.071
Protein (kg)	0.046	0.060	0.052	0.059	0.050	0.085
Muscularity (pts)	0.022	0.005	0.016	0.002	0.015	-0.022
Udder Conformation (pts)	-0.029	-0.033	-0.029	-0.031	-0.031	-0.041
Udder Volume (pts)	0.173	0.226	0.202	0.187	0.204	0.235
ADG (kg/d)	0.042	0.041	0.041	0.041	0.041	0.039
SEUROP (pts)	1.409	1.307	1.369	1.320	1.375	1.173
Dressing % (points)	0.264	0.237	0.251	0.238	0.258	0.203
SCS (points)	0.262	0.255	0.232	0.244	0.256	0.202

[1]Loose H.=Loose Housing; [2]TMR=Total Mixed Ration; 3 p.=points; [4]TS=Tie-Stall; LH=Loose Housing; Trad=Traditional; TMR=Total Mixed Ration; Yes=Occurrence of pasture: yes; No=Occurrence of pasture: no

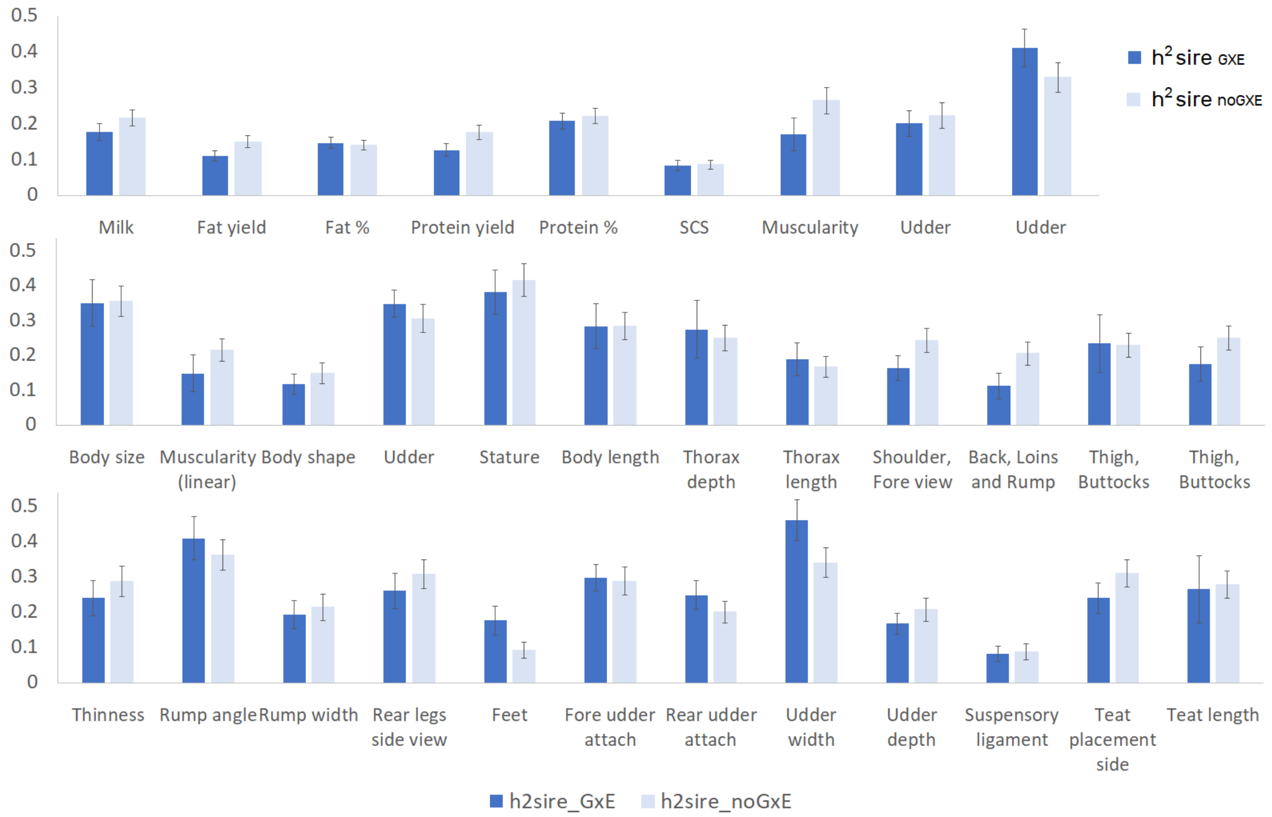

Supplementary Figure S1. Sire heritability obtained including GxE for traits considered in the study. Standard deviation is reported as error bar. All estimates were significantly ($P \leq 0.01$) different from zero (z-scores test).

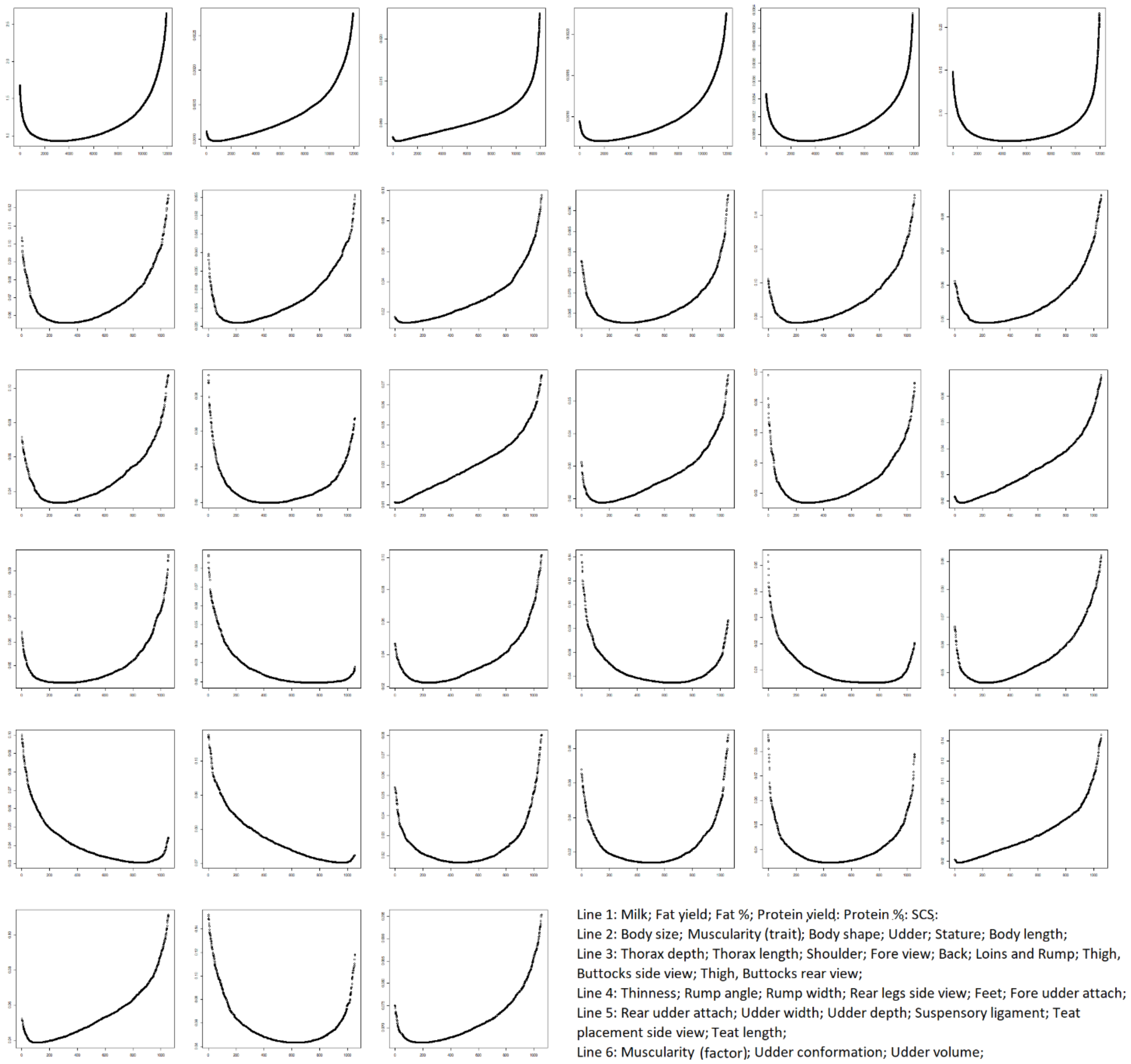

Supplementary Figure S2. Plot of **ZGZ'** variance estimates for traits under study as gradient of variation along the reaction norm for the levels of Herd-Environmental Group effect (HEG), that are the environmental covariate. Names of traits are reported in Figure and refer to the corresponding line of the picture.

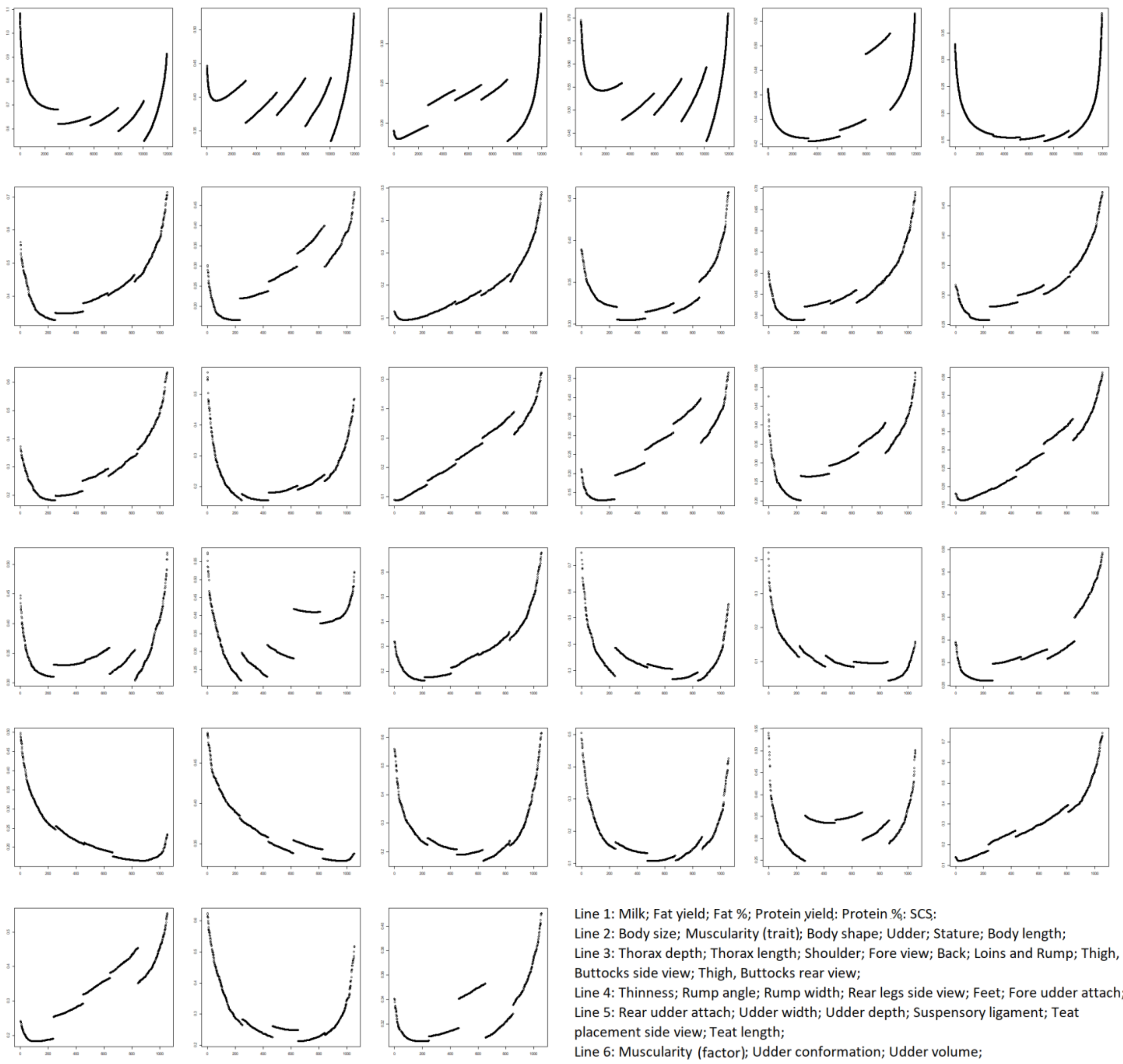

Supplementary Figure S3. Plot of sire model heritability estimates for traits under study as gradient of variation along the reaction norm for the levels of the Herd-Environmental Group effect (HEG), that are the environmental covariate. The discontinuities in the lines are due to the 5 classes of residual variance considered. Names of traits are reported in Figure and refer to the corresponding line of the picture. Sire model heritability of traits was calculated as $h^2 = 4 \cdot \sigma^2_s / \sigma^2_P$, where σ^2_s is the sire variance, and σ^2_P the total phenotypic variance.

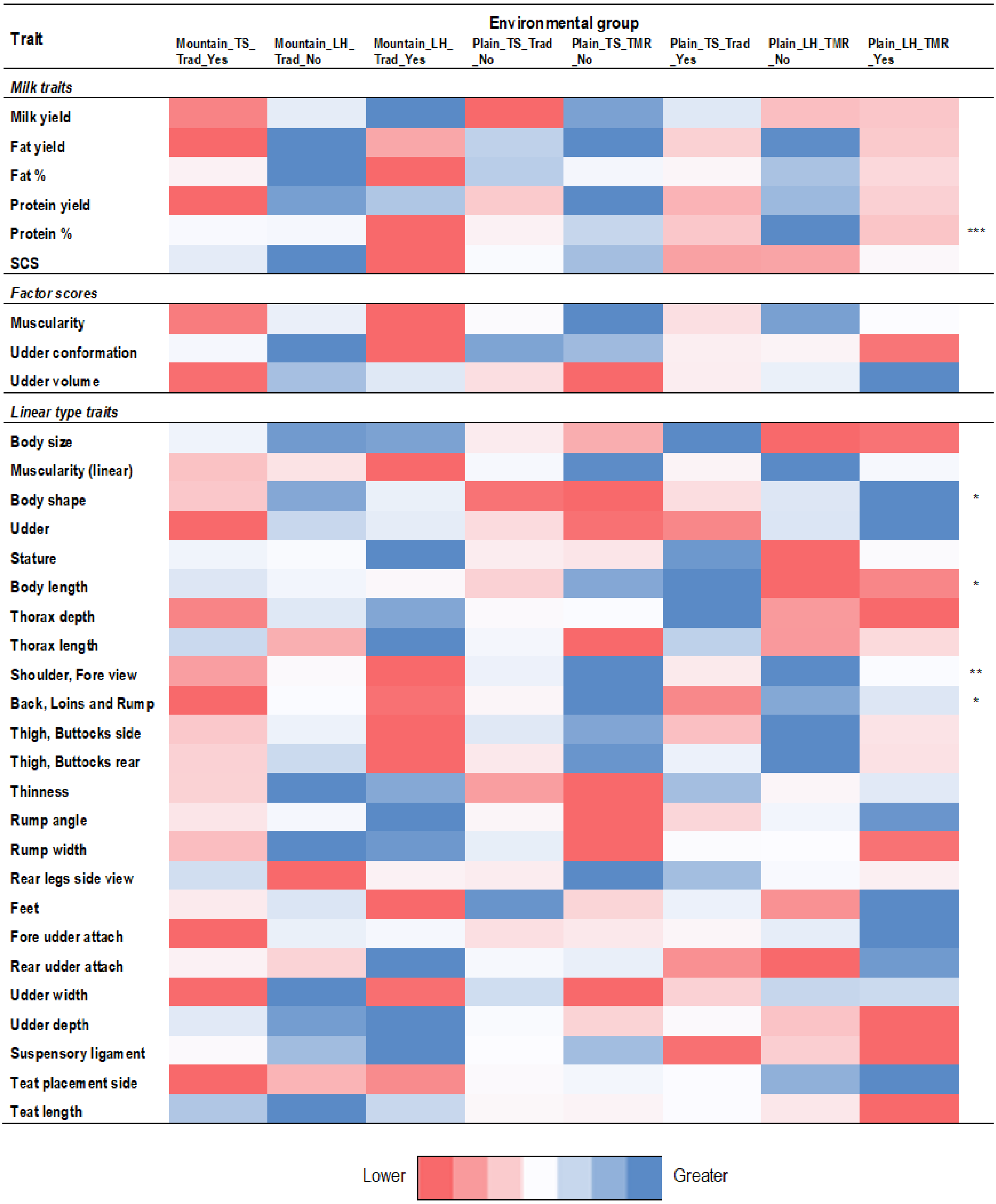

Supplementary Figure S4. Least square means of **ZGZ'** variance of linear type traits in different levels of the environmental groups considered in the study. Values of variance are shown as gradient of colors (red=lower values; blue=higher values). The gradient has to be seen within trait (that is, line by line). The significance of differences among the levels of the same environmental group has been tested via GLM analysis. TS=Tie Stall; LH=Loose Housing; Trad=Traditional; TMR=Total Mixed Ration; Yes=Occurrence of pasture: yes; No=Occurrence of pasture: no; *= P≤0.005; **= P≤0.01: ***= P≤0.001
